# What Do We Know About Teamwork in Chinese Hospitals? A Systematic Review

**DOI:** 10.3389/fpubh.2021.735754

**Published:** 2021-12-17

**Authors:** Hujie Wang, Martina Buljac-Samardzic, Wenxing Wang, Jeroen van Wijngaarden, Shasha Yuan, Joris van de Klundert

**Affiliations:** ^1^Erasmus School of Health Policy and Management, Erasmus University Rotterdam, Rotterdam, Netherlands; ^2^Institute of Medical Information and Library, Chinese Academy of Medical Sciences and Peking Union Medical College, Beijing, China; ^3^Prince Mohammad Bin Salman College of Business and Entrepreneurship, King Abdullah Economic City, Saudi Arabia

**Keywords:** teamwork, team performance, team intervention, multidisciplinary team, Chinese hospitals

## Abstract

**Background and Objective:** Improving quality of care is one of the primary goals in current Chinese hospital reforms. Teamwork can play an essential role. Characteristics of teamwork and interventions for improving teamwork in hospitals have been widely studied. However, most of these studies are from a Western context; evidence from China is scarce. Because of the contextual differences between China and Western countries, empirical evidence on teamwork from Western hospitals may have limited validity in China. This systematic review aims to advance the evidence base and understanding of teamwork in Chinese hospitals.

**Methods:** Both English (i.e., Embase, Medline, and Web of Science) and Chinese databases (i.e., CNKI, CQVIP, and Wanfang) were searched for relevant articles until February 6, 2020. We included the studies that empirically researched teamwork in Chinese hospitals. Studies were excluded if they (1) were not conducted in hospitals in Mainland China, (2) did not research teamwork on team interventions, (3) were not empirical, (4) were not written in English or Chinese, (5) were not published in peer-reviewed journals, and (6) were not conducted in teams that provide direct patient care. Both deductive and inductive approaches were used to analyze data. The Mixed Methods Appraisal Tool (MMAT) was used to assess their methodological quality.

**Results:** A total of 70 articles (i.e., 39 English articles and 31 Chinese articles) were included. The results are presented in two main categories: Teamwork components and Team interventions. The evidence regarding the relationships among inputs, processes, and outcomes is scarce and mostly inconclusive. The only conclusive evidence shows that females perceive better team processes than males. Similar types of training and tools were introduced as can be found in Western literature, all showing positive effects. In line with the Chinese health reforms, many of the intervention studies regard the introduction of multidisciplinary teams (MDTs). The evidence on the implementation of MDTs reveals that they have led to lower complication rates, shorter hospital stays, higher diagnosis accuracy, efficiency improvement, and a variety of better disease-specific clinical outcomes. Evidence on the effect on patient survival is inconclusive.

**Conclusion:** The Chinese studies on teamwork components mainly focus on the input-process relationship. The evidence provided on this relationship is, however, mostly inconclusive. The intervention studies in Chinese hospitals predominantly focus on patient outcomes rather than organizational and employee outcomes. The introduction of training, tools, and MDTs generally shows promising results. The evidence from primary hospitals and rural areas, which are prioritized in the health reforms, is especially scarce. Advancing the evidence base on teamwork, especially in primary hospitals and rural areas, is needed and can inform policy and management to promote the health reform implementation.

**Systematic Review Registration:** https://www.crd.york.ac.uk/prospero/display_record.php?ID=CRD42020175069, identifier CRD42020175069.

## Introduction

Improving the quality of hospital care has been one of the primary goals of the Chinese national health reforms since 2009 (1). In recent years, the Chinese government has been making efforts to explore strategies to reach this goal. In Western countries, facilitating interdisciplinary communication, collaboration, and teamwork are emphasized in many quality improvement strategies for hospital care (2, 3). The World Bank and the World Health Organization have also recommended China to enhance teamwork within medical teams of hospitals as a managerial practice to promote the delivery of high-quality hospital care (4). However, a systematic scientific understanding of teamwork and its relationship to the quality of hospital care in China is lacking.

Teamwork significantly impacts the quality and safety of care. Failure in teamwork can result in (preventable) medical errors and adverse events (5–8), while improving teamwork is beneficial for the quality of care (9, 10). Numerous literature reviews have considered teamwork and the improvement of teamwork in hospitals (11–14). Some reviews focus on characteristics that are important for teamwork and team performance. For instance, Mickan and Rodger summarize the characteristics of an effective team in hospitals (e.g., suitable leadership, trust, coordination, and communication) and suggest finding a balance between organizational structure and team processes (11). Lemieux-Charles and McGuire have developed an Integrated (Health Care) Team Effectiveness Model (ITEM), showing the relation between team characteristics, team processes, psycho-social traits, and team performance (12). Other reviews focus on interventions to improve teamwork in hospitals. For example, Buljac-Samardzic et al. present an overview of team interventions (i.e., training, tools, (re)design, and program) to improve team effectiveness (13), and Hughes et al. show a positive impact of team training on trainees' reactions to training, learning outcomes, behaviors, and organizational and patient outcomes (14). A solid body of evidence on teamwork in hospitals exists. With few exceptions, however, the studies included in these reviews are from Western countries. For example, only one study from Buljac-Samardzic et al. (13) review is conducted in Mainland China.

Cultural differences between China and Western countries may influence people's behaviors in a team. For instance, Chinese people emphasize collectivism and are more likely to avoid conflict to preserve harmony within their teams, while people from Western countries prefer individualistic values and are prone to debate with their teammates when disagreement emerges (15, 16). Tjosvold et al. (17) provide empirical evidence showing that collectivism has a positive effect on constructive controversy, which in turn positively influences the performance of teams in Chinese factories. Hui et al. (18) provide evidence of the positive relationship between collectivism and team performance. These examples suggest that teams in Chinese hospitals function differently from those in Western hospitals, which may subsequently translate into differences regarding characteristics of teamwork and the effectiveness of interventions. In other words, the empirical evidence on teamwork from Western hospital settings may have limited validity in a Chinese setting. With the aim to advance the scientific evidence base and understanding of teamwork in Chinese hospitals, we conducted a systematic review to address the following research question: What is the present empirically based knowledge on teamwork in Chinese hospitals?

## Methodology

This systematic review was conducted based on the Preferred Reporting Items for Systematic Reviews and Meta-Analysis (PRISMA) statement (19, 20). The review protocol was registered in PROSPERO (No. CRD42020175069).

### Search Strategy

English and Chinese databases were searched for published articles, not restraining the year of publication. A medical librarian from the Erasmus Medical Center developed the English query, which consisted of keywords that combined three areas: (1) teamwork or team interventions (e.g., teamwork, team performance, team effectiveness, multidisciplinary team, and team training); (2) hospital setting (e.g., hospital and healthcare); and (3) China (i.e., China, Chinese, and the names of the 31 administrative regions in Mainland China). This query was searched in Embase, Medline and Web of Science on February 6, 2020. A Chinese medical librarian assisted in translating the English query and finalizing the Chinese query (both the English and Chinese queries are shown in Supplementary File 1). The Chinese databases CNKI, CQVIP, and Wanfang were searched for articles until February 6, 2020. Finally, 1,533 records were retrieved after all the duplicates deleted: 996 from English databases and 537 from Chinese databases.

### Inclusion and Exclusion Criteria

Based on the research question, we aimed at including studies that empirically researched teamwork in Chinese hospitals. The following exclusion criteria were established: (1) studies that were not conducted in hospitals located in Mainland China; (2) studies that do not provide information about teamwork or team interventions; (3) non-empirical studies, such as editorial letters and literature reviews; (4) articles that are not written in English or Chinese; (5) articles that are not published in peer-reviewed journals, such as conference papers and dissertations; and (6) studies conducted in departments that do not provide direct patient care, such as pharmacy, laboratory, administration, logistics and information technology.

### Selection Process

**Figure 1:**
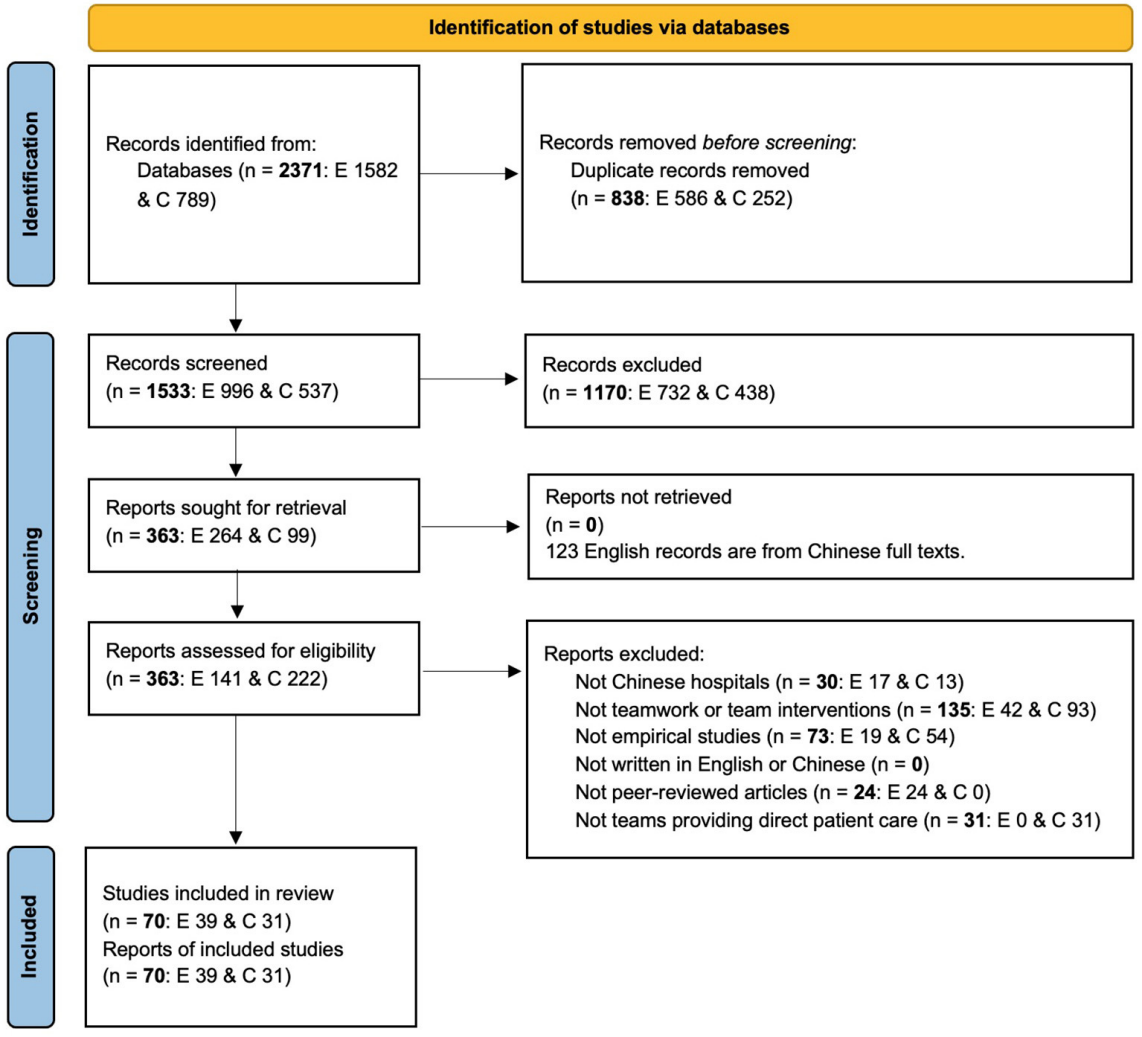
PRISMA flow diagram. E, English; C, Chinese.

There were two stages of selecting articles. Each stage consisted of an English and a Chinese part. Firstly, the titles and abstracts retrieved from both the English and Chinese databases were independently screened by two researchers according to the above-mentioned exclusion criteria. In case of disagreement between the two researchers, consensus would be reached through discussion. In case of any doubt, it was transferred to the second stage. This first stage resulted in a selection of 363 articles (from the 1,533): 264 from English databases and 99 from Chinese databases. Surprisingly, 123 out of the 264 articles with English titles and abstracts are actually written in Chinese. Hence, the numbers of articles written in English and Chinese were adjusted to 141 and 222, respectively. Secondly, the full texts of the 363 articles were independently reviewed by the same researchers of the first stage. In case of disagreement, a third researcher would settle it. Finally, 70 articles (i.e., 39 English articles and 31 Chinese articles) were included for data synthesis. Figure 1 shows the screening and reviewing process based on the PRISMA Flow Diagram (20).

### Data Synthesis

The conducted analysis combined deductive and inductive elements and consisted of four steps.

As a first step, we extracted data from the included articles in terms of author (year), research aim, setting, administrative regions, research methods, time period, main focus of teams considered, team interventions considered (if any), findings related to teamwork (if any), other findings, potentially relevant information from the discussion section, interpretation specifically relevant to the Chinese context, and conclusion. These data fields were selected to systematically extract all information relevant to our research question. In this step, the data from the Chinese articles were translated into English.

The second step combined deductive and inductive approaches to create primary result categories (21). The classical (Western) reviews (12, 13, 22, 23) served as deductive starting points for the categorization process. We used the categories of the ITEM model, which describes team inputs, processes, and outcomes in health care, as well as their interrelations (12). In addition, we included categories identified by systematic reviews on teamwork components (i.e., inputs, processes, and outcomes) in intensive care and chronic care (22, 23).

Combining the categorization in these reviews (deduction) with an initial inductive analysis of the data collected, we identified the input element “team composition” as a first primary category and added articles that research the composition of teams in hospitals to this primary category.

Buljac-Samardzic et al. summarize the interventions implemented in health care teams and categorize the interventions as training, tools, (re)design, and program (13). The second primary category “team interventions” was established based on this review and consisted of articles reporting on interventions on teams in hospitals.

Two additional primary categories were inductively formed to classify the remaining articles. The category “describing teamwork” included descriptive studies reporting on teamwork via questionnaires, interviews, or both. The category “the influence of teamwork on performance” consisted of articles addressing the influence of teamwork on team performances.

In the third step, these primary categories were repeatedly adjusted based on discussions among all authors. The category “team interventions” remained unchanged, while “team composition” was divided into two parts. The first part was composed of studies that actually examine the relationship between the three teamwork components (i.e., inputs, processes, and outcomes) (12, 22, 23). Articles in the primary categories “describing teamwork” and “the influence of teamwork on performance” also research the three teamwork components and the relationships between them. Therefore, these two primary categories were merged with the first “team composition” category, forming a new category “teamwork components” (see also 12, 22, 23). The second part of “team composition” consisted of studies that research interventions on team composition (i.e., (re)design and program) and was added to the category “team interventions.” These adjustments resulted in the two final categories “teamwork components” and “team interventions.”

In step four, the two categories were further divided into several subcategories according to the theoretical frameworks and reviews mentioned above (12, 13, 22, 23). The first category “teamwork components” was divided into processes, relationship between inputs and processes, relationship between inputs and outcomes, and relationship between processes and outcomes based on the teamwork theoretical models (12, 22, 23). The second category “team interventions” included training, tools, (re)design and program, in accordance with the categorization of Buljac-Samardzic et al. (13) review. Table 1 shows the categorization of results and the number of articles per category and subcategory.

**Table 1:** Categorization of results.

**Main category**	**Subcategories**	**Number of articles**
Teamwork components:		25^*^
	Processes	4
	Relationship between inputs and processes	16
	Relationship between inputs and outcomes	4
	Relationship between processes and outcomes	3
Team interventions:		45
	Training	6
	Tools	3
	(Re)design	20
	Program	16
Total		70

* *Two studies researched two kinds of relationships each, thus the total number of studies in the four subcategories exceeds the number of studies of the category “teamwork components”*.

### Quality Assessment

The Mixed Methods Appraisal Tool (MMAT) was used to assess the methodological quality of the included studies (24). The quality score of a study, ranging from 0 to 5, was the number of criteria a study met. All the studies were divided into high quality (scoring 4 or 5) and low quality (scoring 3 or less) studies (25).

## Results

### Overall Findings

Most studies in the first category address relationships across the three components of the input-process-outcome framework. The second category describes the specific interventions implemented and their effects on outcomes. More than 70% of the studies were conducted in tertiary hospitals. With one exception, all studies were situated in urban hospitals. In the following paragraphs, we summarize the main findings of the review. Supplementary Tables 1, 2 provide a complete overview of the results.

Based on the MMAT scores, the majority of the studies (60 out of 70 studies) are of high methodological quality, while the other ten studies are of low quality in the research design. The quality of research design of each study is also shown in both Supplementary Tables 1, 2.

### Teamwork Components

#### Processes

Collaboration is one of the process elements of the ITEM model (12) and, two out of the four studies in this subcategory focus on collaboration (26, 27). Sharing the same goal is one of the strategies that facilitate the collaboration within a team (26), while lack of common ground is a barrier to the collaboration between healthcare professionals (27). The other two studies measure team processes with two well-known patient safety culture questionnaires: the Hospital Survey on Patient Safety Culture (HSOPSC) (i.e., “teamwork within units,” “teamwork across units,” and “communication openness”) and the Safety Attitudes Questionnaire (SAQ) (i.e., “teamwork climate”) (28–31). One of these two studies compare results between Chinese and US hospitals, showing significantly higher scores of “teamwork within units” and “teamwork across units” but significantly lower scores of “communication openness” in the Chinese hospital (29).

#### Relationship Between Inputs and Processes

Sixteen studies explore the relationship between inputs and processes (32–47). The majority of the articles in this subcategory are based on HSOPSC and SAQ (10 out of 16) (33, 35–37, 39, 40, 42, 44–46). The input “gender” is found to influence team processes. Female staff perceive significantly better “communication openness” (42), “teamwork within units” (46), and “teamwork climate” (45) than male staff. The relationship between the input “profession” and team processes is inconclusive, although profession is researched the most in these studies. Two HSOPSC studies show that nurses score “communication openness” significantly higher than doctors (37, 42), while two other HSOPSC studies find no significant differences between the ratings of doctors and nurses (36, 46). Two SAQ studies find that doctors evaluate “teamwork climate” significantly more positively than nurses (35, 45).

Mixed results are also found in terms of education level and age. Staff with a degree higher than bachelor score “communication openness” (42) and “teamwork across units” (46) significantly higher but “teamwork climate” significantly lower than those with an education level lower than bachelor (35, 45). Staff younger than 25 years report significantly higher scores for “teamwork climate” than those older than 50 years in one study (45) but the opposite is found in another study (35). Besides, two HSOPSC studies compare the results between China and the US without testing significance, showing that overall Chinese healthcare professionals score higher in the three process related composites than their counterparts in the US (37, 42), except for “teamwork across units” in one study (42).

Five out of the six remaining studies investigate the input-process relationship via other questionnaires (32, 34, 38, 41, 47). Similar to the findings of the previous HSOPSC and SAQ studies, female doctors perceive significantly better team interaction (e.g., communication, coordination, and mutual help) than male doctors (47). Profession, department, and age also influence healthcare professionals' ratings on team processes. The overall teamwork scores of internal medicine nurses are significantly lower than those of surgical nurses (34, 38). However, internal medicine doctors score team interaction significantly higher than surgeons (47). Staff younger than 30 years perceive better overall teamwork than those older than 30 years in one study (38) but score team cohesion significantly lower than those between 40 and 50 years old in another study (41). In addition, cultural values are considered to affect team processes (43). Feminine traits (e.g., friendship, enthusiasm, and patience) are shown to be beneficial to communication; collectivism facilitates the mutual support, while a clique culture hinders it.

#### Relationship Between Inputs and Outcomes

Four studies examine the correlation between inputs and outcomes (48–51). Disciplinary diversity shows positive effects on team performance (i.e., the number of team consultations) (50). Tenure and team size are found to influence team outcomes. Staff working between 16 and 30 years perceive significantly worse job satisfaction than other staff (49), while nurses working more than 20 years report significantly more adverse events than those working less than 20 years (51). Adding additional members to a stable surgical team increases the surgical procedure time (48).

#### Relationship Between Processes and Outcomes

Three studies investigate the process-outcome relationship (47, 51, 52). Teamwork is a positive predictor to nurses' adverse events reporting (51) but is negatively related to nurses' willingness to make plans for their retirement (52). All the six factors of team interaction (i.e., communication, coordination, mutual help, team goals, work norms, and cohesion and conflict resolution) are inversely related to physicians' burn-out (47).

### Team Interventions

#### Training

Training as a team intervention focuses on enhancing inputs and team processes, consequently resulting in improved outcomes. Most studies on training evaluate simulation-based training. Simulation, the core of simulation-based training, refers to “a technique to replace or amplify real-patient experiences with guided experiences, artificially contrived, that evokes or replicates substantial aspects of the real world in a fully interactive manner” (53). All the five studies on simulation-based training are conducted in emergency settings (e.g., trauma care, pediatric septic shock, cardiac surgeries, and advanced cardiac life support) (54–58). The forms of simulated scenarios include mannequins (55), simulators (56), and animals (58). Two studies find the inputs (e.g., surgical skills and emergency skills) significantly improved after the training (57, 58), while two other studies observe significantly better outcomes (e.g., task complete compliance and work efficiency) in the simulation group, compared to the non-simulation group (56) or pre-intervention group (54). One study concludes that licensed perfusionists score communication and coordination higher than the trainees in a cardiac surgery simulation scenario, without testing significance (55). In addition to the studies on simulation-based training, there is one study on TeamSTEPPS (i.e., Team Strategies and Tools to Enhance Performance and Patient Safety). TeamSTEPPS is a training system aiming at improving healthcare professionals' teamwork and communication skills (inputs), facilitating information sharing, resolving conflicts (processes), and finally providing better patient care (outcomes) (59). This study on TeamSTEPPS presents descriptive results that more healthcare professionals rate their communication skills as good after the training (60).

#### Tools

Tools in this subcategory refer to SBAR (i.e., Situation-Background-Assessment-Recommendation tool) and checklists, both aiming at optimizing the team processes. SBAR is a structured template used to facilitate the communication between team members (61). Two studies have evaluated SBAR and show significantly better patients' and healthcare professionals' satisfaction, and a significant decrease in the incidence of adverse events (62, 63). Moreover, one of these two studies also shows higher work efficiency (62). A checklist is a list of actions to be done in a hospital setting, with the goal of avoiding any steps being forgotten (64). Yuan et al. (65) have implemented a self-developed electronic checklist for multidisciplinary team meetings and report significantly higher working efficiency and diagnosis accuracy and lower hospital stay but no significant change in terms of the incidence of complications.

#### (Re)Design

(Re)design is defined as constructing or revising the input characteristics and/or the processes of a medical team within hospitals.

Multidisciplinary teams (MDTs) are the main focus of most studies in this subcategory (18 out of 20 studies) (66–83). An MDT is a team consisting of healthcare professionals from different disciplines that work together to provide better patient care (84). Five studies describe or evaluate the effects of establishing MDTs (revising the inputs) in cancer (66, 67, 80), trauma (82), and stroke care (78). Significantly higher diagnosis accuracy and lower incidence of complications and hospital stay are reported in these studies (66, 80, 82). Eight studies implement MDTs with clarified roles and responsibilities of team members (defining the inputs) (69–73, 75, 77, 81), which results in significantly higher quality of life and patients' satisfaction and lower incidence of complications. The other five studies on MDT consider the standardization and optimization of the working procedures of MDTs (optimizing team processes) through a pathway of care (79), a new procedure (68, 76, 83) or re-organizing multidisciplinary meetings (74). The results of these studies are significantly higher overall survival rate, shorter hospital stay, less complications, and better disease-specific clinical outcomes. In addition to the outcomes reported above, two studies present mixed results regarding hospitalization costs (68, 76), and two other studies find no significant changes in mortality rate (76, 82). Moreover, four out of the eighteen studies only summarize the outcomes after the (re)design, without controls (70, 71, 78, 83).

Of the remaining two studies, one clarifies roles and responsibilities of a non-MDT (85) and reports significantly higher nursing quality and patients' satisfaction. The other study optimizes the working procedures of medical teams via a novel team performance appraisal system (86). Per capita performance and healthcare professionals' satisfaction are significantly higher, but the overall patients' satisfaction is significantly lower in the experiment group compared to those in the control group.

#### Program

A fixed component of programs is (re)design, which is combined with training, a tool, or both. MDTs are also involved in 7 out of the 16 studies on program (87–93). Nine studies combine (re)design with training on technical skills (inputs) (87–89, 94–99). The outcomes are significantly higher patients' satisfaction, nursing quality, and working efficiency, and lower incidence of medical errors. Notable, two studies show lower incidence of complications and higher work efficiency, without testing significance (97, 99). Four studies evaluate programs that combine (re)design with rounds (90–93), a structured tool referring to a group of healthcare professionals meeting around a patient to discuss the patient's condition (13). Three out of these four studies present significantly lower incidence of complication and hospital stay and decreased depression scores (90–92), while one study only summarizes the results (93). Lastly, three studies introduce programs in which all the three types of interventions are combined for postoperative care (100, 101) or cancer pain care (102). One study reports a reduction in complications and no significant change in recovery time (100). Another study shows significant pain reduction (102), while the third study reports a sustainable significantly increase in the teamwork score (101).

## Discussion

This systematic review presents an overview of research on teamwork in Chinese hospitals. We first summarize the findings of the relationships among the three teamwork components (i.e., inputs, processes, and outcomes) and then list the evidence on interventions to improve teamwork and achieve better team outcomes. As more than 70% of the studies were conducted in tertiary hospitals and nearly all the studied hospitals are in urban areas, the evidence base on primary and secondary hospitals and hospitals in rural areas is very limited.

More than half of the studies that research teamwork components focus on the relationship between inputs (e.g., age, gender, profession, education level, and department) and processes (e.g., teamwork within units, teamwork across units, and teamwork climate). This relationship has received little attention in Western literature so far (12, 103).

Despite the relatively large number of studies on the input-process relationship included in our review, the evidence synthesis is inhibited by the heterogeneity of variables used, the mixed results, and the primary research goals that are not focused on this relationship. The only conclusive evidence that can be synthesized from the review findings is that females perceived better team processes (i.e., communication openness, teamwork within units, teamwork climate, and team interaction) than males. This may be explained by the differences in personality traits between females and males. Females have been reported to be more agreeable than males, which means that females are more willing to cooperate and maintain harmony (104, 105). The evidence on the relationships between other inputs and processes is inconclusive.

Six studies research the input-outcome relationships, process-outcome relationships, or both (47–52). These studies, however, focus on different input, process, and outcome variables, which makes it difficult to synthesize the results across studies. One study shows a positive correlation between disciplinary diversity (input) and the number of team consultations (outcome) (50). Another study shows that better team interaction as a process variable (e.g., communication, coordination, and cohesion) is associated with less burn-out (outcome), suggesting a positive influence of team interaction on team performance (47). These results are in line with Lemieux-Charles and McGuire's review that most of the inputs (e.g., disciplinary diversity) and processes (e.g., communication, coordination, and cohesion) have positive correlations with team outcomes (12). Altogether, however, the evidence on the input-outcome and process-outcome relationships is still scarce. More studies are needed to strengthen the evidence on the relationships of outcomes with processes and inputs.

The studied trainings and tools correspond to those mentioned in Western literature (13). The three studies on efficiency all present evidence of improvement (54, 56, 62). Moreover, two studies on SBAR report higher patients' satisfaction (62, 63), and two studies report improved technical skills as an effect of training (57, 58). These results are in line with the findings of Buljac-Samardzic et al. (13) that most trainings and tools result in improvements in team performance. However, the evidence base on training and tools identified in our review is still small.

As was the case for the studies on team components, many team intervention studies regard multidisciplinary teams. The World Bank and the World Health Organization have recommended forming MDTs to promote people-centered integrated care and the quality of care, both of which play important roles in the Chinese health reforms (4). Correspondingly, there has been much research emphasis on MDT implementation in Chinese hospitals, which contrasts with the findings of Buljac-Samardzic et al. (13) review on team interventions. We find consistent evidence that MDTs are associated with reduced complication rates and length of hospital stays, and improved efficiency and diagnostic accuracy (66, 75–77, 79, 88–91). Nine of the MDT studies present better disease-specific clinical outcomes for different conditions (67–69, 73, 75, 80, 81, 87, 92). These findings support the positive effects of MDTs, which is in line with the findings of Western MDT studies (106, 107).

The evidence on the effects of MDT implementation on survival is inconclusive. Three studies report higher survival rates (67, 74, 80), while two other studies find no significant change in mortality rates after MDT implementation (76, 82). This inconclusive finding may be explained by the different severity, treatment, and prognosis of the diseases researched in these studies.

Kirkpatrick (108) divides the team training evaluation into four levels: reactions (e.g., people's reactions and feedbacks to the intervention), learning (e.g., knowledge and skills learnt), behavior (e.g., participants' behavioral change at work), and results (e.g., patient outcomes and organizational outcomes). Based on these four levels, the majority of the included intervention studies in our review focus on patient outcomes which belong to the fourth level (i.e., results). However, studies in Western reviews commonly regard the first three levels (i.e., reactions, learning, and behavior) and organizational outcomes which are a part of the fourth level (13). This difference may be due to the different research aims of Chinese and Western studies and different research interests of researchers from China and Western countries.

Advocating harmony and collectivism are typical Chinese cultural values, which differ from Western countries (15, 16). Three studies comparing the results between China and the US show higher scores on “teamwork within units” in Chinese hospitals but mixed results on “teamwork across units” and “communication openness” (29, 37, 42), proposing the value attached to the harmony in the Chinese culture as an explanation. Another Chinese cultural trait, collectivism, is reported to promote mutual support (43). These findings and inferences form first evidence on teamwork in China in relation to cultural differences with Western countries.

### Limitation

This review has several limitations. Firstly, books and gray literature were not included. Secondly, the translation of the query from English to Chinese may have led to missing results in Chinese databases. With the assistance of a Chinese librarian, the two queries have been made as equivalent as possible. Thirdly, because of publication bias, intervention studies which have not produced desired results may have been underreported. Finally, although we assessed the methodological quality, the included studies are heterogenous, making it difficult to synthesize the evidence. This limits the certainty of evidence of our findings.

### Implications for Future Research

Firstly, patient outcomes have been predominant in the teamwork research in China, while important team outcomes such as healthcare professionals' satisfaction and well-being have received little attention. The team outcomes deserve future research to advance the evidence base on team performance, as is conducive to designing, selecting, and assessing team interventions.

Secondly, the evidence base on the relationships among the three teamwork components deserves strengthening. The included studies seldomly aim to investigate these relationships, causing the evidence on the relationships among inputs, processes, and outcomes to be largely inconclusive. More appropriately designed studies addressing these relationships are called for, as they will also promote the understanding of interventions on inputs (e.g., the introduction of MDTs) related to processes and subsequently to organizational outcomes and patient outcomes.

Lastly, it is important to recognize that China is a large country with considerably variety across provinces (109). The impact of this variety of contexts (e.g., different cultures) on teamwork and team performance is largely unexplored. Most studies are from tertiary hospitals in urban China. Due to the contextual differences, it cannot be assumed that this evidence has validity in lower-level hospitals and rural areas. In view of the priority attached to improving primary care and rural healthcare in the Chinese health reforms (110), valid evidence for primary hospitals and rural China is urgently called for.

## Conclusion

The Chinese studies on teamwork components mainly focus on the input-process relationship. The evidence provided on this relationship is, however, mostly inconclusive. The intervention studies in Chinese hospitals predominantly focus on patient outcomes rather than organizational and employee outcomes. The introduction of training, tools, and MDTs generally shows promising results. The evidence from primary hospitals and rural areas, which are prioritized in the health reforms, is especially scarce. Advancing the evidence base on teamwork, especially in primary hospitals and rural areas, is needed and can inform policy and management to promote the health reform implementation.

## Data Availability Statement

The raw data supporting the conclusions of this article will be made available by the authors, without undue reservation.

## Author Contributions

SY provided support in finalizing the Chinese search query. HW and MB-S screened the English titles and abstracts. HW and WW screened the Chinese titles and abstracts. HW, MB-S, and JK reviewed the English full texts. HW, WW, and SY reviewed the Chinese full texts. HW, MB-S, JW, and JK analyzed the data and categorized the results together. HW initiated the draft of the manuscript and revised it based on the inputs of MB-S, JW, and JK. All authors read and approved the final manuscript.

## Funding

This work was supported by China Scholarship Council (No. 201906160092; receiver: HW). This funder has no role in the study design, data collection and analysis, interpretation of data, and writing the manuscript.

## Conflict of Interest

The authors declare that the research was conducted in the absence of any commercial or financial relationships that could be construed as a potential conflict of interest.

## Publisher's Note

All claims expressed in this article are solely those of the authors and do not necessarily represent those of their affiliated organizations, or those of the publisher, the editors and the reviewers. Any product that may be evaluated in this article, or claim that may be made by its manufacturer, is not guaranteed or endorsed by the publisher.

## Supplementary Material

Supplementary File 1 English and Chinese search queries.

Supplementary File 2 PRISMA 2020 checklist.

Supplementary File 3 PRISMA 2020 abstract checklist.

Supplementary File 4 MMAT assessment.

Supplementary Table 1 Summary of results (teamwork component).

Supplementary Table 2 Summary of results (team interventions).

## Abbreviations

MMAT: Mixed Methods Appraisal Tool
MDTs: multidisciplinary teams
ITEM: Integrated (Health Care) Team Effectiveness Model
PRISMA: Preferred Reporting Items for Systematic Reviews and Meta-Analysis
HSOPSC: Hospital Survey on Patient Safety Culture
SAQ: Safety Attitudes Questionnaire
TeamSTEPPS: Team Strategies and Tools to Enhance Performance and Patient Safety
SBAR: Situation-Background-Assessment-Recommendation.
