# Supplementary material for: What Do We Know About Teamwork in Chinese Hospitals? A Systematic Review
Source: Front Public Health. 2021 Dec 17;9:735754. doi: 10.3389/fpubh.2021.735754 (PMC8719585; doi:10.3389/fpubh.2021.735754)
Supplement: Supplementary File 1 — English and Chinese search queries. [file Data_Sheet_1.docx]

**English and Chinese search queries**

**1. English query**

***(1) embase.com***

('teamwork'/exp OR 'team building'/de OR 'collaborative care team'/de OR 'multidisciplinary team'/de OR 'team nursing'/de OR 'crew resource management'/de OR ('group process'/de AND ('health care personnel'/exp OR hospital/exp OR 'nursing'/exp OR 'health care'/exp)) OR (teamwork* OR team-work* OR team-building* OR teambuilding* OR teamtraining* OR team-training* OR (team NEAR/6 (cooperation* OR perform* OR function* OR effectiv* OR interaction* OR communication* OR interdiscipl* OR multidiscipl* OR inter-discipl* OR multi-discipl* OR improve* OR enhance* OR qualit* OR safe*)) OR sbar OR Situation-Background-Assessment-Recommendation):ab,ti) AND ('China'/de OR 'Guangxi'/de OR 'Inner Mongolia'/de OR 'Ningxia'/de OR 'Tibet'/de OR 'Xinjiang'/de OR 'Chinese'/de OR (China OR (Chinese NEAR/3 (hospital* OR institute* OR nursing)) OR Anhui OR Beijing OR Chongqing OR Fujian OR Gansu OR Guangdong OR Guangxi-Zhuang OR Guizhou OR Hainan OR Hebei OR Heilongjiang OR Henan OR Hubei OR Hunan OR Inner-Mongolia OR Jiangsu OR Jiangxi OR Jilin OR Liaoning OR Ningxia-Hui OR Qinghai OR Shaanxi OR Shandong OR Shanghai OR Shanxi OR Sichuan OR Tianjin OR Tibet OR Xinjiang-Uyghur OR Yunnan OR Zhejiang):ab,ti OR (china OR Chinese):jt OR [Chinese]/lim)

***(2) Medline ALL ovid***

(Crew Resource Management, Healthcare/ OR (Group Processes/ AND (exp Health Personnel/ OR exp Hospitals/ OR Nursing/ OR health care/)) OR (teamwork* OR team-work* OR team-building* OR teambuilding* OR teamtraining* OR team-training* OR (team ADJ6 (cooperation* OR perform* OR function* OR effectiv* OR interaction* OR communication* OR interdiscipl* OR multidiscipl* OR inter-discipl* OR multi-discipl* OR improve* OR enhance* OR qualit* OR safe*)) OR sbar OR Situation-Background-Assessment-Recommendation).ab,ti.) AND (China/ OR Beijing/ OR Tibet/ OR (China OR (Chinese ADJ3 (hospital* OR institute* OR nursing)) OR Anhui OR Beijing OR Chongqing OR Fujian OR Gansu OR Guangdong OR Guangxi-Zhuang OR Guizhou OR Hainan OR Hebei OR Heilongjiang OR Henan OR Hubei OR Hunan OR Inner-Mongolia OR Jiangsu OR Jiangxi OR Jilin OR Liaoning OR Ningxia-Hui OR Qinghai OR Shaanxi OR Shandong OR Shanghai OR Shanxi OR Sichuan OR Tianjin OR Tibet OR Xinjiang-Uyghur OR Yunnan OR Zhejiang).ab,ti. OR (china OR Chinese).jw. OR Chinese.la.)

***(3) Web of Science***

TS=(((teamwork* OR team-work* OR team-building* OR teambuilding* OR teamtraining* OR team-training* OR (team NEAR/5 (cooperation* OR perform* OR function* OR effectiv* OR interaction* OR communication* OR interdiscipl* OR multidiscipl* OR inter-discipl* OR multi-discipl* OR improve* OR enhance* OR qualit* OR safe*)) OR sbar OR Situation-Background-Assessment-Recommendation)) AND ((China OR (Chinese NEAR/2 (hospital* OR institute* OR nursing)) OR Anhui OR Beijing OR Chongqing OR Fujian OR Gansu OR Guangdong OR Guangxi-Zhuang OR Guizhou OR Hainan OR Hebei OR Heilongjiang OR Henan OR Hubei OR Hunan OR Inner-Mongolia OR Jiangsu OR Jiangxi OR Jilin OR Liaoning OR Ningxia-Hui OR Qinghai OR Shaanxi OR Shandong OR Shanghai OR Shanxi OR Sichuan OR Tianjin OR Tibet OR Xinjiang-Uyghur OR Yunnan OR Zhejiang)) AND (hospital*))

**2. Chinese query**

***(1) CNKI***

**TI**=("团队"+"多学科"+"跨学科"+"学科间")*("培训"+"合作"+"协作"+"协调"+"沟通"+"交流"+"效果"+"绩效"+"表现"+"质量"+"安全")*("医院"+"医疗机构"+"初级卫生保健"+"卫生院"+"社区卫生服务中心"+"社区卫生服务机构") **OR** **KY**=("团队"+"多学科"+"跨学科"+"学科间")*("培训"+"合作"+"协作"+"协调"+"表现"+"交流"+"效果"+"沟通"+"绩效"+"质量"+"安全")*("医院"+"医疗机构"+"初级卫生保健"+"卫生院"+"社区卫生服务中心"+"社区卫生服务机构")

***(2) CQVIP***

**M**=("团队" OR "多学科" OR "跨学科" OR "学科间") AND ("培训" OR "合作" OR "协作" OR "协调" OR "沟通" OR "交流" OR "效果" OR "绩效" OR "表现" OR "质量" OR "安全") AND ("医院" OR "医疗机构" OR "初级卫生保健" OR "卫生院" OR "社区卫生服务中心" OR "社区卫生服务机构")

***(3) WANFANG (Only for journals)***

**题名或关键词**:(("团队" OR "多学科" OR "跨学科" OR "学科间") AND ("培训" OR "合作" OR "协作" OR "协调" OR "沟通" OR "交流" OR "效果" OR "绩效" OR "表现" OR "质量" OR "安全") AND ("医院" OR "医疗机构" OR "初级卫生保健" OR "卫生院" OR "社区卫生服务中心" OR "社区卫生服务机构"))
